# Glycocalyx biomarkers as early predictors of endotheliopathy in pediatric and young adult hematopoietic stem cell transplantation patients

**DOI:** 10.3389/fonc.2026.1789000

**Published:** 2026-05-08

**Authors:** Kimberly Uchida, Kris M Mahadeo, Jennifer McArthur, Yvonne Avent, Chia-Wei Hsu, Haitao Pan, Lama Elbahlawan, Melissa Hines, Akshay Sharma, Ruth G. Tatevossian, Sebabrata Mahapatra, Meenasri Kumbaji, Irtiza Sheikh, Ying Li, Keri Schadler, Saad Ghafoor

**Affiliations:** 1 Department of Pediatric Medicine, Division of Critical Care Medicine, St. Jude Children’s Research Hospital, Memphis, TN, United States; 2 Department of Pediatrics, Division of Pediatric Critical Care Medicine, The University of Tennessee Health Science Center, Memphis, TN, United States; 3 Department of Pediatrics, Division of Pediatric Critical Care, University of Utah Health, Salt Lake, UT, United States; 4 Department of Pediatrics, Division of Pediatric Transplantation and Cellular Therapy, Duke University, Durham, NC, United States; 5 Department of Biostatistics, St. Jude Children's Research Hospital, Memphis, TN, United States; 6 Department of Bone Marrow Transplantation & Cellular Therapy, St. Jude Children's Research Hospital, Memphis, TN, United States; 7 Clinical Biomarkers Laboratory, Department of Pathology, St. Jude Children’s Research Hospital, Memphis, TN, United States; 8 Department of Stem Cell Transplantation, Division of Pediatrics, The University of Texas MD Anderson Cancer Center, Houston, TX, United States; 9 Department of Pediatrics Research, Division of Pediatrics, The University of Texas MD Anderson Cancer Center, Houston, TX, United States

**Keywords:** angiopoietin-2 (Ang-2), children and young adults, endotheliopathy, hematopoietic stem cell transplant (HSCT), IGFBP-3, syndecan-1, TIMP-1

## Abstract

**Introduction:**

Discovery of biomarkers predictive of endotheliopathy after hematopoietic stem cell transplant (HSCT) has the potential to more rapidly identify vulnerable patients, guide treatment decisions, track responses to therapy, and inform targets for novel treatment. This study aimed to evaluate the levels and predictive capabilities of glycocalyx biomarkers for endotheliopathy in the first 100 days following HSCT in pediatric and young adult patients.

**Methods:**

Parallel prospective observational pilot studies were performed to examine markers related to endothelial glycocalyx structure, function, and regulation at St. Jude Children’s Research Hospital (SJCRH) and MD Anderson Cancer Center (MDACC). The MDACC study took a discovery-driven approach using a 55-biomarker panel performed at two early timepoints whereas the SJCRH study adopted a targeted approach evaluating a 6-biomarker panel at eight scheduled timepoints. Biomarker levels in patients’ post-HSCT who did or did not develop endotheliopathy were compared at specific timepoints predicative value was assessed using Area Under the Curve (AUC) and Youden Index.

**Results:**

In the SJCRH study, Syndecan-1 and Angiopoietin-2 levels were found to be significantly different between cases of endotheliopathy and controls at multiple time points. Predictive values for both were good to excellent when comparing the time-point immediately before diagnosis of endotheliopathy in cases to standardized time-points of Days 7, 14, and 21 in controls. This was noted for endotheliopathy in general as well for sinusoidal obstruction syndrome (SOS)/veno-occlusive disease (VOD) or transplant-associated thrombotic microangiopathy (TA-TMA). In the MDACC study, a signature of markers was found to be associated with endotheliopathy on Days 0 and 7. Specifically, Tissue inhibitor of metalloproteinases-1 (TIMP-1) and insulin-like growth factor-binding protein-3 (IGFBP-3) had perfect performance (AUC of 1) for endotheliopathy.

**Discussion:**

Biomarkers of glycocalyx damage and dysregulation have significant potential to predict endotheliopathy in pediatric and young adult patients undergoing HSCT. The convergence of these two studies, one targeted and one unbiased, affirms the central role of endothelial injury in post-HSCT complications and provides a list of potential candidate predictors. Given small sample size and hence limited generalizability, further research is required to validate these findings in larger patient cohorts.

## 1 Introduction

Pediatric and young adult patients undergoing hematopoietic stem cell transplantation (HSCT) remain at high risk for complications and mortality (1). Endotheliopathy (injury and dysfunction of the endothelium) contributes to many of the severe complications of HSCT, such as capillary leak syndrome (CLS), engraftment syndrome (ES), sinusoidal obstructive syndrome (SOS)/veno-occlusive disease (VOD), transplant-associated thrombotic microangiopathy (TA-TMA), graft-versus-host disease (GVHD), idiopathic pneumonia syndrome (IPS), and diffuse alveolar hemorrhage (DAH) (2–7). In addition to these specific post-HSCT endotheliopathy syndromes, children who become critically ill also experience endothelial dysfunction (8, 9).

The pathophysiology of endotheliopathy following HSCT is complex and can result from pre-existing pathology, treatments related to primary disease HSCT indication, the pre-HSCT conditioning regimen, various drugs used during and after transplantation, interactions between the host and graft, infectious insults, and/or a combination of these factors (3, 4, 6). Endotheliopathy is often a systemic process and can lead to inappropriate extravasation of cells and fluid into the extravascular space, coagulopathy, cytokine dysregulation, and impaired microvascular blood flow (3, 8, 10). Prompt recognition and treatment of HSCT-related endotheliopathy are crucial to prevent impaired graft function, delayed hematopoietic recovery, and organ failure (8, 10). No single laboratory test has been found to be clinically predictive of developing endotheliopathy, nor of predicting disease course once HSCT complications have occurred. Accordingly, there is a significant unmet need and interest to further characterize biomarkers for this purpose (2, 4, 8, 11–13). We aimed to study biomarkers related to the structure, function, and regulation of the endothelial glycocalyx. The glycocalyx is a thin layer of the vascular endothelium that is highly dynamic and its dysregulation has been implicated in several disease processes such as sepsis and atherosclerosis (14, 15). The glycocalyx is mostly comprised of proteoglycans, glycoproteins, and plasma proteins. It is continuously degraded and replaced, and performs complex physiological roles while interfacing with blood such as mediating vascular permeability, flow of blood, and inflammation (14–19). The biomarkers selected in the St. Jude Children’s Research Hospital Study included a custom panel of commercially available assays including angiopoietin-2 (Ang-2), E-Selectin, intercellular adhesion molecule 3 (ICAM-3), P-Selectin, and thrombomodulin. Angiopoietin-2 is a mediator of glycocalyx breakdown and has been implicated in SOS/VOD and GVHD (7, 20, 21). E- and P-selectin are glycoproteins that mediate leukocyte interactions with the endothelium (16). ICAM-3 is an intercellular adhesion molecule expressed on leukocytes and some endothelial cells, and contributes to inflammation (22, 23). Syndecan-1 is a key structural proteoglycan of the endothelial glycocalyx (13, 14, 17, 18). Thrombomodulin is an endothelial protein with anticoagulant properties (16) and loss of thrombomodulin is thought to contribute to endothelial vulnerability (7). The MD Anderson Cancer Center Study used a commercially available human angiogenesis kit in order to explore biomarkers related to endothelial structure and function in an unbiased fashion. Non-invasive, easy to obtain biomarkers of endotheliopathy predictive of HSCT morbidity could identify at-risk patients, guide the decision to initiate treatment, track the response to therapy, as well as inform potential targets for novel treatment.

We hypothesized in two parallel prospective studies that the levels of these biomarkers would be more severely altered in patients who later developed endotheliopathy, compared to those who did not, at specified time points following HSCT. We also aimed to assess the predictive value of these markers for the development of endotheliopathy, and specifically SOS/VOD and TA-TMA, after HSCT. The two studies taken together broaden our understanding of the role of the endothelium in HSCT complications, and therefore are reported together.

## 2 Materials and methods

Parallel prospective observational pilot studies were performed at St. Jude Children’s Research Hospital from November 2022 to January 2024 and at MD Anderson Cancer Center from June 2021 to December 2022 to examine biomarkers related to the structure and function of the glycocalyx as biomarkers of endotheliopathy in pediatric and young adult patients during the first 100 days after HSCT.

### 2.1 Patient eligibility and study enrollment

Institutional Review Board (IRB) approvals were obtained at each site prior to enrolling patients. For both studies, the inclusion criteria were age of 26 years or younger and that the patient was planning to undergo HSCT. Exclusion criteria were weight less than 10 kg at the time of informed consent, if 5 mL blood samples drawn at the specified intervals posed more than minimal risk, or if there was lack of agreement from the primary HSCT physician to participate in the study. Clinical data were recorded in a secure research electronic database.

### 2.2 Blood sample collection

This was a non-interventional study that required blood sample collection. For the MD Anderson Cancer Center Study, blood samples were collected at Day 0 and Day 7. For the St. Jude Children’s Research Hospital study, patients had a minimum of eight scheduled blood samples collected (Pre-conditioning and Days 0, 1, 7, 14, 21, 28, and 100) after HSCT. If an individual patient received tandem transplants, samples were collected only during the first transplant. Samples were stored in a de-identified biorepository. Once the Day 100 sample was collected, the patient’s participation in the study ended.

### 2.3 Diagnostic criteria/definitions

For both studies, all enrolled patients were screened multiple times weekly for endotheliopathy (CLS, DAH, ES, gastrointestinal or liver GVHD, IPS, SOS/VOD, TA-TMA). For St. Jude Children’s Research Hospital Analysis A (see section 2.5), all unplanned PICU admissions were included in one of the endotheliopathy analysis groups, as critical illness is a known risk factor for endothelial dysfunction (8, 9). Patients were also considered to have endotheliopathy if there was biopsy-proven disease, the clinician documented the diagnosis in the medical record, or the study team found that previously accepted criteria were met. For diagnosis of CLS, ES, and GVHD, the European Society for Blood and Marrow Transplantation criteria were used (24). For diagnosis of SOS/VOD, the modified European Society for Blood and Bone Marrow Transplantation was used (25). For diagnosis of TA-TMA, the modified Jodele criteria were used (26). In patients with GVHD, only those with involvement of an organ system other than the skin were counted as endotheliopathy patients in the study. This was because we anticipated many patients would have non-clinically significant skin GVHD.

### 2.4 Laboratory analysis

Samples obtained at St. Jude Children’s Research Hospital Study were tested from 250 μL citrate plasma samples stored at -80 °C. Samples were thawed, aliquoted, and refrozen at -80 °C. All biomarker testing was performed after samples underwent two freeze-thaw cycles. A custom U-plex 6-plex plate-based electrochemiluminescence immunoassay platform from Meso Scale Discovery (MSD) was used to measure Syndecan-1, E-Selectin, ICAM-3, P-Selectin, thrombomodulin, and Angiopoietin-2 (Ang-2). All assays were performed following the manufacturer’s instructions, samples were run at least in duplicate, and concentrations of analytes were interpolated from standard curves. Intra- and inter-assay coefficients of variation were calculated from the assay results.

For the MD Anderson Cancer Center Study, blood was collected in citrate buffer, plasma was isolated by centrifugation, and stored in 250 μl aliquots at -80°C. Biomarker testing was performed after one freeze-thaw cycle. The Proteome Profiler Human Angiogenesis Array Kit (Bio-techne R&D Systems, catalog ARY007) was used for semi-quantitative analysis. Assays were performed following the manufacturer’s instructions with one sample per membrane, with each factor measured in duplicate for each sample. Relative intensity for each factor was determined by densitometry using Image J (NIH) and normalized against controls within and between each membrane. The biomarker level was then reported as a relative intensity compared to the internal control (which was set to 1).

### 2.5 Statistical analysis

For the St. Jude Children’s Research Hospital study, two analyses were performed. In Analysis A, any patient with endotheliopathy (CLS, DAH, ES, gastrointestinal or liver GVHD, IPS, SOS/VOD, TA-TMA, or PICU admission) within the study period was included as a case. This was done to evaluate the ability of biomarkers to distinguish patients who did or did not develop endotheliopathy more generally. In Analysis B, only patients who developed SOS/VOD or TA-TMA were analyzed as cases. This was in order to identify if any biomarkers were more specific to these endotheliopathies that tend to come with a high level of morbidity and mortality, for which early therapy is known to improve outcomes (26–33). For the MD Anderson Cancer Center Study, patients with any endotheliopathy were analyzed as cases and were then compared to controls. Only one analysis was performed as the patient group with either SOS/VOD or TA-TMA were the same as the patient group that developed any endotheliopathy.

For both studies, continuous biomarker levels were summarized using appropriate descriptive statistics and compared at each time point. Between-group comparisons were performed using Wilcoxon tests due to non-normal distributions. Given the exploratory nature of this pilot study, these time-point specific comparisons were descriptive and intended to identify potential temporal patterns.

For the St. Jude Research Hospital Study, cases were evaluated at the time point immediately preceding the clinical diagnosis of endotheliopathy, while controls were evaluated at the fixed time-point of Day 14 as this time point represents an early post-transplant period during which most patients were clinically stable and had available samples across the cohort. Comparisons were made between the entire cohort of cases and the entire cohort of controls. Sensitivity analysis was performed for the surrounding time points of Days 7 and 21 for controls. Of note, if multiple endotheliopathies occurred in a single patient, the first event that occurred temporally was used. For the MD Anderson Cancer Center Study, direct Day 0 control to Day 0 case and Day 7 control to Day 7 case comparisons were done.

Discriminatory performance of biomarkers was evaluated using receiver operating characteristic (ROC) analysis, with area under the curve (AUC) and optimal cutoffs values for the various biomarkers explored by the Youden index. All tests were two-sided, and a P-value <0.05 was considered statistically significant. Given the exploratory nature of this pilot study, no formal adjustment for multiple comparisons was applied. Statistical analyses were performed using R (4.4.0).

## 3 Results

### 3.1 St. Jude children’s research hospital study results

#### 3.1.1 Patient characteristics

A total of 31 patients were included in the St. Jude Children’s Research Hospital study. Patient characteristics by analysis group are displayed in **Table 1**. Baseline demographic and transplant-related characteristics were generally comparable between cases and controls in both Analysis A and Analysis B, although formal comparisons were limited by small sample size (**Table 1**). Individual patient characteristics and timing of endotheliopathy events are provided in**Supplementary Table 1**. All but one patient survived the entire study period. Seventeen patients had endotheliopathy (CLS, DAH, ES, gastrointestinal or liver GVHD, IPS, SOS/VOD, TA-TMA, or PICU admission) with six patients developing either SOS/VOD or TA-TMA. Seven patients required PICU care, with septic shock and respiratory failure being the most common reasons for requiring admission to the PICU.

**Table 1:** Baseline demographic and transplant characteristics of St. Jude Children’s Research Hospital patients, stratified by endotheliopathy status (Analysis A) and by SOS/VOD or TA-TMA status (Analysis B).

Characteristic		All patients (N = 31)	Analysis A			Analysis B		
			Control (N = 14)	Case of endotheliopathy (N = 17)	P value	Control (N = 25)	Case of SOS/VOD or TA-TMA (N = 6)	P value
Age in years at transplant Median (range)		11 (2–22)	13 (4–22)	9 (2-18)	0.1764	12.5 (2-22)	10 (2-18)	0.8217
Sex	Female	13 (41.9%)	6 (42.9%)	7 (41.2%)	0.9248	10 (40%)	3 (50%)	0.6758
	Male	18 (58.1%)	8 (57.1%)	10 (58.8%)		15 (60%)	3 (50%)	
Prior Transplant	No	26 (83.9%)	12 (85.7%)	14 (82.4%)	1.0000	21 (84%)	5 (83.3%)	1.0000
	Yes	5 (16.1%)	2 (14.3%)	3 (17.6%)		4 (16%)	1 (16.7%)	
Transplant Indication	Leukemia/Lymphoma	21 (67.7%)	9 (64.3%)	12 (70.6%)	0.8603	16 (64%)	5 (83.3%)	1.0000
	Non-malignant hematologic disorder	7 (22.6%)	4 (28.6%)	3 (17.6%)		6 (24%)	1 (16.7%)	
	Solid tumor	3 (9.7%)	1 (7.1%)	2 (11.8%)		3 (12%)	0 (0%)	
Donor Type	Autologous	3 (9.7%)	1 (7.1%)	2 (11.8%)	0.9422	3 (12%)	0 (0%)	0.6567
	Matched sibling	3 (9.7%)	1 (7.1%)	2 (11.8%)		2 (8%)	1 (16.7%)	
	Matched unrelated	7 (22.6%)	4 (28.6%)	3 (17.6%)		5 (20%)	2 (33.3%)	
	Mismatched related	18 (58.1%)	8 (57.1%)	10 (58.8%)		15 (60%)	3 (50%)	
Graft Type	Bone marrow	8 (25.8%)	5 (35.7%)	3 (17.6%)	0.4125	6 (24%)	2 (33.3%)	0.6344
	Peripheral blood	23 (74.2%)	9 (64.3%)	14 (82.4%)		19 (76%)	4 (66.7%)	
Conditioning Type	Myeloablative	9 (29%)	4 (28.6%)	5 (29.4%)	0.8324	6 (24%)	3 (50%)	0.4586
	Non-myeloablative	1 (3.2%)	1 (7.1%)	0 (0%)		1 (4%)	0 (0%)	
	Reduced-intensity	21 (67.7%)	9 (64.3%)	12 (70.6%)		18 (72%)	3 (50%)	
T-cell depletion agent	Alemtuzumab	1 (3.2%)	1 (7.1%)	0 (0%)	0.5757	1 (4%)	0 (0%)	1.0000
	Anti-thymocyte globulin (ATG)	19 (61.3%)	9 (64.3%)	10 (58.8%)		15 (60%)	4 (66.7%)	
	N/A	11 (35.5%)	4 (28.6%)	7 (41.2%)		9 (36%)	2 (33.3%)	
Endotheliopathy	CLS	5 (16.1%)	0 (0%)	5 (29.4%)		4 (16%)	1 (16.7%)	1.0000
	Critical illness	7 (22.6%)	0 (0%)	7 (41.1%)		4 (16%)	3 (50%)	0.1100
	DAH	1 (3.2%)	0 (0%)	1 (5.9%)		0 (0%)	1 (16.7%)	0.1935
	ES	5 (16.1%)	0 (0%)	5 (29.4%)		4 (16%)	1 (16.7%)	1.0000
	GI GVHD	2 (6.5%)	0 (0%)	2 (11.8%)		1 (4%)	1 (16.7%)	0.3548
	SOS/VOD	4 (12.9%)	0 (0%)	4 (23.5%)		0 (0%)	4 (66.7%)	0.0005
	TA-TMA	5 (16.1%)	0 (0%)	5 (29.4%)		0 (0%)	5 (83.3%)	<0.001
Survived study period	No	1 (3.2%)	0 (0%)	1 (5.9%)	1.0000	0 (0%)	1 (16.7%)	0.1935
	Yes	30 (96.8%)	14 (100%)	16 (94.1%)		25 (100%)	5 (83.3%)	

Capillary leak syndrome (CLS), diffuse alveolar hemorrhage (DAH), engraftment syndrome (ES), gastrointestinal graft versus host disease (GI GVHD), sinusoidal obstruction syndrome/veno-occlusive disease (SOS/VOD), thrombotic microangiopathy (TA-TMA).

#### 3.1.2 Median biomarker levels by time-point

**Figure 1:**
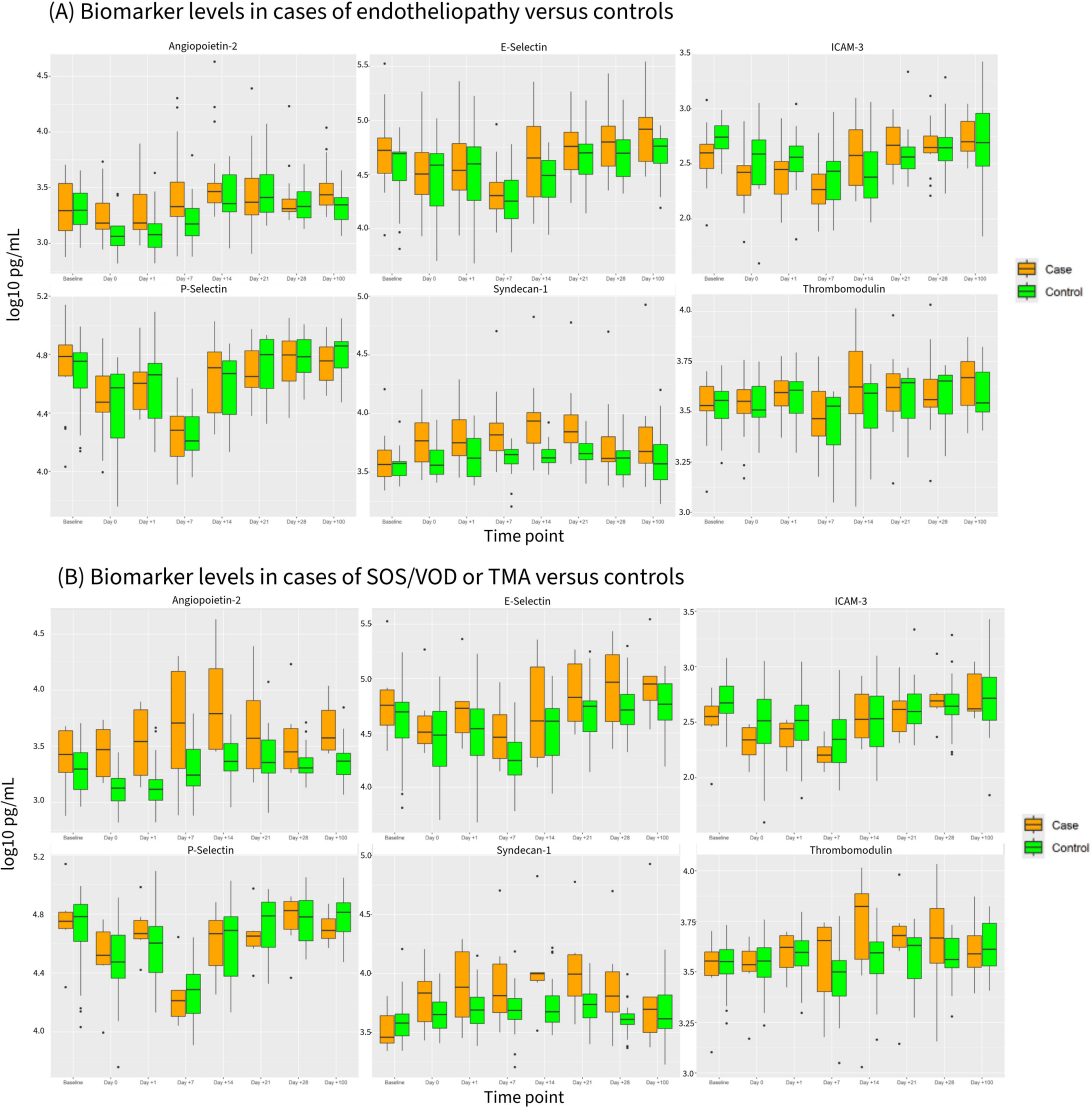
Biomarker levels for St. Jude Children's Research Hospital Study at each time-point in cases of endotheliopathy **(A)** or SOS/VOD or TA-TMA **(B)** versus controls. The center line represents the median and the box represents the interquartile range. Whiskers show biomarker levels within 1.5 times the interquartile range and individual points are outliers. The figure shows log10 transformation of biomarker levels in pg/mL.

Median biomarker levels by time-point in controls versus cases of all endotheliopathy (Analysis A) or cases of SOS/VOD or TA-TMA (Analysis B) are displayed in **Figure 1**. For Analysis A (endotheliopathy vs. controls), a statistically significant difference in biomarker levels between cases and controls was observed for Syndecan-1 at Days 0, 1, 7, 14, and 21, and for Ang-2 at Days 1 and 7 (p<0.05). For Analysis B (SOS/VOD or TA-TMA only vs. controls), this difference was observed for Syndecan-1 at Day 21 and for Ang-2 at Days 0, 1, 14, 100 (p<0.05).

#### 3.1.3 Biomarker levels at the time-point preceding endotheliopathy diagnosis compared with day 14 in controls

**Figure 2:**
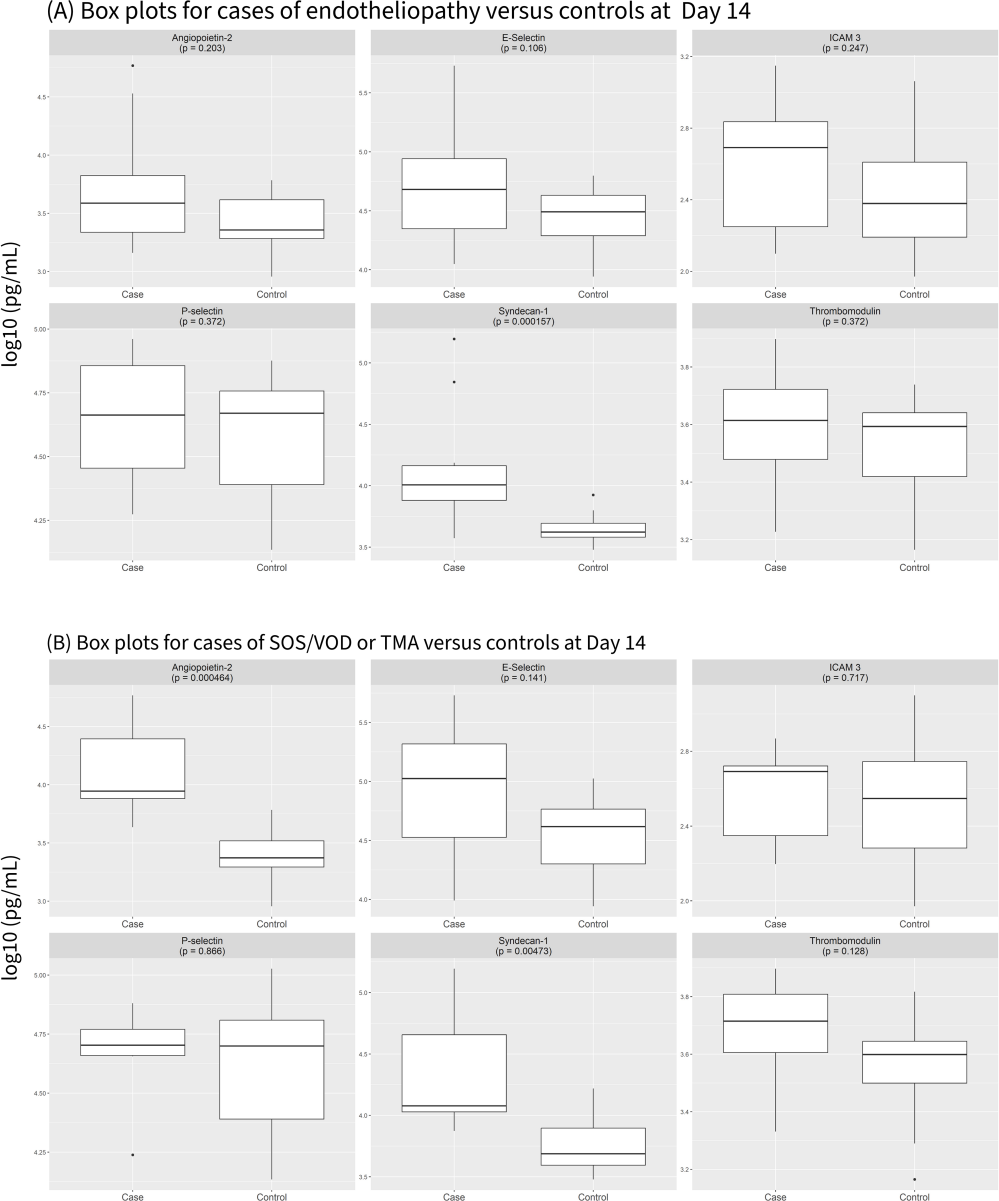
Biomarker levels in St. Jude Children's Research Hospital Study immediately preceding endotheliopathy diagnosis **(A)** or SOS/VOD or TA-TMA diagnosis **(B)** compared to Day 14 in controls. The center line represents the median and the box represents the interquartile range. Whiskers show biomarker levels within 1.5 times the interquartile range and individual points are outliers. For cases, the time-point immediately preceding the diagnosis of either SOS/VOD or TA-TMA was used. For controls, the standardized time-point of Day 14 was used. The figure shows log10 transformation of biomarker levels in pg/mL.

Boxplots comparing the time-point in cases immediately preceding diagnosis of any endotheliopathy (Analysis A) or either SOS/VOD or TA-TMA (Analysis B) to Day 14 in controls are shown in **Figure 2**. For cases, biomarker levels correspond do the measurement obtained immediately preceding clinical diagnosis of endotheliopathy. For controls, levels at Day 14 are shown. Syndecan-1 was significantly elevated in cases compared to controls in both analyses (Analysis A: P<0.001, Analysis B: P = 0.005). Sensitivity analyses using Day 7 and Day 21 in controls as alternative time-point comparisons yielded consistent results, with Syndecan-1 remaining significantly elevated in cases compared to controls in both analyses (**Supplementary Figure 1**). In both analyses, all the biomarkers studied except ICAM-3 were significantly elevated in cases compared to controls at the Day 7 time-point. However, in Analysis A, Syndecan-1 was the only biomarker that consistently distinguished cases from controls when Days 14 and 21 were used as control time-points. (**Figure 2**, **Supplementary Figure 1**). Ang-2 was significantly elevated in the cases of either SOS/VOD or TA-TMA compared to the controls at Day 14 (P<0.001) in Analysis B (**Figure 2**). Sensitivity analyses using Day 7 and Day 21 in controls as alternative time-point comparisons yielded consistent results (**Supplementary Figure 1**).

#### 3.1.4 Biomarker performance by AUC and exploratory optimal cut-off values

Biomarker discrimination performance assessed by AUC and exploratory optimal cut-off/threshold values using the Youden Index are shown in **Table 2** for Analysis A and **Table 3** for Analysis B. Using Day 14 as the control time-point, Syndecan-1 demonstrated good predictive performance in both analyses (Analysis A: AUC = 0.897, Analysis B: AUC = 0.872). An exploratory threshold of 6894.44 pg/mL yielded a sensitivity of 93% and a specificity of 78% for developing endotheliopathy (Analysis A) and an exploratory threshold of 6983.83 pg/mL yielded 73% sensitivity and 100% specificity for developing either SOS/VOD or TA-TMA. Sensitivity analyses using Days 7 and 21 as alternative control time-points demonstrated consistent AUC estimates for Syndecan-1 in both analyses with similar optimal cut-off/threshold values. Notably, when Day 21 was used as the control time-point in Analysis A, the corresponding threshold achieved perfect sensitivity within this pilot cohort. In Analysis B, Ang-2 demonstrated excellent discriminatory performance (AUC = 0.968) for SOS/VOD and TA-TMA using Day 14 as the control time-point. Sensitivity analyses using Days 7 and 21 yielded similarly high AUC values, although the corresponding optimal cut-off values varied across time points.

**Table 2:** Biomarker discrimination performance by AUC and exploratory optimal threshold for St. Jude Children's Research Hospital Study Analysis A.

Biomarker	Day 14		Day 7		Day 21	
	AUC	Threshold Youden Index (pg/mL)	AUC	Threshold Youden Index (pg/mL)	AUC	Threshold Youden Index (pg/mL)
Angiopoietin-2	0.635	2857.15	0.837	3208.69	0.632	2838.27
E-Selectin	0.671	64546.82	0.782	41439.25	0.47	30862.1
ICAM-3	0.623	444.43	0.675	420.34	0.534	468.92
P-Selectin	0.405	47755.32	0.917	38493.79	0.603	60861.91
Syndecan-1	0.897	6894.44	0.905	6807.13	0.868	6967.42
Thrombomodulin	0.595	4789.02	0.738	4005.52	0.453	4290.99

The time-point immediately before diagnosis of any endotheliopathy in the case group was compared to Day 14 in controls. Sensitivity analysis was performed using Days 7 and 21 in controls as the comparison time-point.

**Table 3:** Biomarker discrimination performance by AUC and exploratory optimal threshold for St. Jude Children's Research Hospital Study Analysis B.

Biomarker	Day 14		Day 7		Day 21	
	AUC	Threshold Youden Index (pg/mL)	AUC	Threshold Youden Index (pg/mL)	AUC	Threshold Youden Index (pg/mL)
Angiopoietin-2	0.968	6807.47	0.987	3940.36	0.949	4235.94
E-Selectin	0.699	129874.07	0.807	66604.01	0.667	71319.23
ICAM 3	0.551	478.47	0.733	452.22	0.507	475.65
P-selectin	0.526	45022.08	0.913	44643.06	0.580	60861.91
Syndecan-1	0.878	6983.83	0.947	7030.69	0.957	7248.95
Thrombomodulin	0.705	4975.22	0.820	4549.40	0.717	5021.24

Time-point immediately before diagnosis of only SOS/VOD and/or TA-TMA in case group was compared to Day 14 in controls. Sensitivity analysis was performed using Days 7 and Day 21 in controls as the comparison time-point.

### 3.2 MD Anderson cancer center results

#### 3.2.1 MD Anderson cancer center patient characteristics

A total of 14 patients were included in the MD Anderson Cancer Center study. Baseline demographic and transplant-related characteristics for the MD Anderson Cancer Center study are demonstrated in **Table 4**. Individual patient characteristics are shown in **Supplementary Table 2**. There were differences between the endotheliopathy group and the control group with regard to age, donor type, graft type, and T-cell depletion agent. Nine patients did not develop any post-HSCT endotheliopathy and 5 patients developed at least one endotheliopathy within the first 100 days post-HSCT. One of the 14 patients died before the end of the study period, and one patient who developed SOS/VOD was excluded from analysis due to failed quality control of the biomarker assay. Of the patients who developed endotheliopathy were included in the analysis, two had SOS/VOD diagnoses within the first 7 days (within the period for which blood was analyzed) and two developed SOS/VOD within the week following Day 7. Three of the patients who developed SOS/VOD developed at least one additional endotheliopathy (TA-TMA, GI GVHD, or ICU admission).

**Table 4:** Baseline demographic and transplant characteristics of MD Anderson Cancer Center study patients.

Characteristic		All patients (N = 13)	Control (N = 9)	Case of SOS/VOD (N = 4)	P value
Age in years at transplant Median (range)		10 (2-25)	21 (6-25)	7 (2-20)	0.0443
Sex	Female	6 (46.1%)	5 (55.5%)	1 (25%)	0.5594
	Male	7 (58.1%)	4 (44.4%)	3 (75%)	
Prior Transplant	No	12 (92.3%)	8 (88.9%)	4 (100%)	1.0000
	Yes	1 (7.7%)	1 (11.1%)	0 (0%)	
Transplant Indication	Leukemia/Lymphoma	11 (84.6%)	8 (88.9%)	3 (75%)	0.5385
	Non-malignant hematologic disorder	0 (0%)	0 (0%)	0 (0%)	
	Solid tumor	1 (7.7%)	1 (11.1%)	0 (0%)	
	Inherited Metabolic Disorder	1 (7.7%)	0 (0%)	1 (25%)	
Donor Type	Autologous	4 (30.7%)	4 (44.4%)	0 (0%)	0.0042
	Matched sibling	2 (15.3%)	2 (22.2%)	0 (0%)	
	Matched unrelated	2 (15.3%)	2 (22.2%)	0 (0%)	
	Mismatched unrelated				
	Mismatched related	1 (7.7%)	1 (11.1%)	0 (0%)	
	Unrelated Cord Blood	4 (30.7%)	0 (0%)	4 (100%)	
Graft Type	Bone marrow	2 (15.4%)	2 (%)	0 (0%)	0.0014
	Peripheral blood	7 (53.8%)	7 (64.3%)	0 (0%)	
	Cord Blood	4 (30.8%)	0 (0%)	4 (100%)	
Conditioning Type	Myeloablative	12 (92%)	8 (89%)	4 (100%)	1.0000
	Non-myeloablative	0 (0%)	0 (0%)	0 (0%)	
	Reduced-intensity	1 (7.7%)	1 (11.1%)	0 (0%)	
T-cell depletion agent	Alemtuzumab	0 (0%)	0 (0%)	0 (0%)	0.0154
	Anti-thymocyte globulin (ATG)	3 (23%)	0 (0%)	3 (75%)	
	Post-transplant cyclophosphamide (PTCY)	5 (38.4%)	5 (55.5%)	0 (0%)	
	N/A	6 (46.1%)	5 (55.5%)	1 (25%)	
Endotheliopathy	CLS	0 (0%)	0 (0%)	0 (0%)	
	Critical illness	3 (23%)	0 (0%)	3 (75%)	
	DAH	1 (7.7%)	0 (0%)	1 (25%)	
	ES	0 (0%)	0 (0%)	0 (0%)	
	GI GVHD	2 (15.4%)	0 (0%)	3 (75%)	
	SOS/VOD	4 (30.8%)	0 (0%)	4 (100%)	
	TA-TMA	1 (7.7%)	0 (0%)	1 (25%)	
Survived study period	No	1 (7.7%)	0 (0%)	1 (25%)	0.3077
	Yes	12 (92.3%)	9 (100%)	3 (75%)	

Capillary leak syndrome (CLS), diffuse alveolar hemorrhage (DAH), engraftment syndrome (ES), gastrointestinal graft versus host disease (GI GVHD), sinusoidal obstruction syndrome/veno-occlusive disease (SOS/VOD), thrombotic microangiopathy (TA-TMA).

#### 3.2.2 Relative biomarker levels in MD Anderson cancer center patients at day 0 and day 7

**Tables 5**, **6** summarize the biomarkers that were significantly (P<0.05) associated with endotheliopathy at Day 0 and Day 7 respectively. Eleven biomarkers showed unadjusted associations with the risk of endotheliopathy at Day 0 in this exploratory analysis. ADAMTS-1, FGF-4, FGF-basic, IGFBP-3, PlGF, and VEGF-C were significantly lower at Day 0 in patients who developed endotheliopathy than in those who did not develop endotheliopathy. IGFBP-1, IGFBP-2, and TIMP-1 were significantly higher at Day 0 in patients who developed endotheliopathy than in control patients. At Day 7, a broad pattern of biomarker differences was observed between patients who did and did not subsequently develop endotheliopathy, with most angiogenic and growth-related factors showing lower relative levels in the endotheliopathy group, and TIMP-1 being the only marker consistently higher in endotheliopathy patients (p = 0.006). At Day 7, fourteen factors were significantly lower in patients who developed endotheliopathy compared to control (p<0.05): Amphiregulin, Angiopoietin-1, Coagulation Factor III, EG-VEGF, FGF-4, FGF-basic, GDNF, GM-CSF, IGFBP-3, MCP-1, PD-ECGF, PDGF-AB/PDGF-BB, uPA, and Vasohibin (**Table 6**).

**Table 5:** Exploratory biomarkers showing unadjusted associations with subsequent endotheliopathy at Day 0 in the MD Anderson Cancer Center Study.

Biomarker	Endotheliopathy	No endotheliopathy	P value
ADAMTS-1	0.30 (0.29-0.38)	0.58 (0.33-0.66)	0.037
FGF basic	0.31 (0.21-0.37)	0.59 (0.33-0.66)	0.037
FGF-4	0.31 (0.21-0.38)	0.59 (0.33-0.68)	0.037
IGFBP-1	0.90 (0.87-0.92)	0.73 (0.34-0.88)	0.017
IGFBP-2	0.95 (0.91-0.98)	0.86 (0.64-0.93)	0.037
IGFBP-3	0.57 (0.54-0.57)	0.85 (0.68-0.99)	0.007
PlGF	0.32 (0.29-0.4)	0.60 (0.33-0.68)	0.037
Thrombospondin-1	0.96 (0.74-1.13)	0.80 (0.68-0.87)	0.045
VEGF-C	0.32 (0.30-0.38)	0.59 (0.33-0.68)	0.037

Values are presented as median, with interquartile range (IQR) in brackets.

**Table 6:** Exploratory biomarkers showing unadjusted associations with subsequent endotheliopathy at Day 7 in the MD Anderson Cancer Center Study.

Biomarker	Endotheliopathy	No endotheliopathy	P-value
Angiopoietin-1	0.32 (0.21-0.34)	0.63 (0.33-0.66)	0.011
Amphiregulin	0.29 (0.21-0.36)	0.63 (0.32-0.65)	0.037
Coagulation Factor III	0.30 (0.21-0.35)	0.62 (0.33-0.64)	0.017
FGF basic	0.28 (0.21-0.35)	0.61 (0.32-0.66)	0.037
FGF-4	0.29 (0.21-0.35)	0.63 (0.33-0.66)	0.017
GDNF	0.32 (0.21-0.35)	0.63 (0.32-065)	0.037
GM-CSF	0.30 (0.22-0.35)	0.63 (0.33-0.65)	0.017
IGFBP-3	0.49 (0.44-0.64)	0.89 (0.67-0.97)	0.007
MCP-1	0.35 (0.22-0.39)	0.64 (0.34-0.69)	0.037
PD-ECGF	0.32 (0.23-0.39)	0.61 (0.36-0.66)	0.017
PDGF-AB/PDGF-BB	0.32 (0.21-0.35)	0.62 (0.32-0.65)	0.025
PlGF	0.30 (0.21-0.38)	0.62 0.32-0.65)	0.045
TIMP-1	1.03 (0.99-1.06)	0.93 (0.74-0.96)	0.007
uPA	0.33 (0.24-0.36)	0.62 (0.32-0.65)	0.037
Vasohibin	0.32 (0.21-0.36)	0.62 (0.32-0.65)	0.037

Values are presented as median, with interquartile range (IQR) in brackets.

#### 3.2.3 MD anderson cancer center study exploratory biomarker discrimination

In these exploratory analyses of the MD Anderson Cancer Center patients, several biomarkers demonstrated strong discriminatory ability for subsequent development of endotheliopathy. At Day 0, IGFBP-1, IGFBP-3, and TIMP-1 showed particularly high AUC values, with IGFBP-3 and TIMP-1 achieving complete separation within this pilot cohort. At Day 7, a broad pattern of strong biomarker discrimination was observed, with multiple biomarkers demonstrating high AUC values (**Table 7**). Notably, IGFBP-3 and TIMP-1 again achieved complete separation at this time point.Exploratory threshold values for all biomarkers using the Youden Index at Day 0 and Day 7 are shownin **Supplementary Tables 3**, **4**. It should be noted that this was a semi-quantitative assay so the thresholds are reported as a relative intensity (see “Methodology”, section 2.4).

**Table 7:** Exploratory biomarkers in the MD Anderson Cancer Center Study demonstrating AUC greater than 0.9 for later development of SOS/VOD on Day 7.

Biomarker	AUC
Angiopoietin-1	0.972
Coagulation Factor III	0.944
EG-VEGF	0.944
FGF-4	0.944
GM-CSF	0.944
IGFBP-3	1
PD-ECGF	0.944
PDGF-AB/PDGF-BB	0.917
TIMP-1	1

## 4 Discussion

This prospective pilot study demonstrated that biomarkers of glycocalyx and endothelial integrity, measured early after HSCT, have strong potential to serve as early indicators of endotheliopathy in pediatric and young adult patients. While E-selectin, P-selectin, and Thrombomodulin exhibited only temporary and modest predictive value, Syndecan-1 and Angiopoietin-2 consistently showed early and significant elevations with promising predictive value in the St. Jude Children’s Research Hospital data, underscoring their promise as candidate clinical biomarkers. Findings from the MD Anderson Cancer Center study support the biological pathways highlighted in the St. Jude Children’s Research Hospital findings.

Results from the St. Jude data strongly support Syndecan-1, a key structural proteoglycan of the endothelial glycocalyx (13, 14, 17, 18), as a central biomarker in HSCT-related endotheliopathy. Shedding of Syndecan-1 is a well-established response to endothelial stress in conditions such as sepsis and septic shock (14). In our study, Syndecan-1 levels were elevated at the time of endotheliopathy diagnosis and remained significantly higher in cases compared to controls at every measured time point from Day 0 through 21, indicating a sustained disturbance of the glycocalyx in high-risk patients. The increase in Syndecan-1 levels preceding clinical diagnosis of endotheliopathy highlights its predictive value rather than serving solely as a diagnostic marker. This is further supported by its strong predictive performance for endotheliopathy when using Day 14 as a comparison in controls (AUC = 0.897), with a cut-off of 6894.44 pg/mL yielding high sensitivity (93%) and specificity (78%). Sensitivity analyses and the ability to predict severe complications, such as SOS/VOD and TA-TMA, using a similar optimal cut-off (6983.83 pg/mL) and perfect specificity within this pilot cohort reinforce the robustness of this finding. These results support and extend existing research. Horan et al. also reported an association between elevated Syndecan-1 and severe SOS/VOD and GVHD in pediatric HSCT (34). Similarly, Seidel et al. demonstrated the utility of Syndecan-1 in predicting acute GVHD when combined with other inflammatory markers (17). Interpreting absolute Syndecan-1 values remains challenging due to the absence of established reference ranges and high assay variability (35). Although the use of a custom research-use-only (RUO) assay limits direct comparison with standardized tests, the consistent results across batches and the clear distinction between cases and controls support the reliability and strong prognostic significance of these findings.

In the St. Jude data, Angiopoietin-2 emerged as an equally strong and potentially more specific predictor of severe endothelial complications, including SOS/VOD and TA-TMA. As a key regulator of vascular destabilization and permeability that promotes glycocalyx breakdown (20, 21), Ang-2 represents a promising biomarker. Ang-2 levels were significantly higher in SOS/VOD and TA-TMA cases at multiple time points. Its predictive accuracy was excellent, with an AUC exceeding 0.9 at the time-point immediately preceding diagnosis when compared to Days 7, 14, and 21 in the control group. Using Day 14 as a comparison in controls, a cut-off of 6807.47 pg/mL achieved perfect sensitivity (100%) and strong specificity (83%). These results corroborate previous findings that elevated Ang-2 levels after HSCT are associated with more severe GVHD and lower event-free survival (36), and increased rates of transplant-related endotheliopathy (37, 38). The established association between high Ang-2 at TA-TMA diagnosis and reduced survival in adults (37) further underscores its prognostic significance. The St. Jude data indicate that Ang-2 elevation occurs very early, signaling the impending severity of vascular injury.

The complementary, discovery-based approach utilized for the MD Anderson Cancer Center data further supports the promise of glycocalyx biomarkers in predicting endotheliopathy complications in pediatric and young adult HSCT patients. Analysis of 55 angiogenesis-related biomarkers in pediatric HSCT patients using direct comparison of levels in cases of endotheliopathy at Days 0 and 7 versus controls identified a robust endotheliopathy risk signature. Ang-2 was not found to be significantly different between cases of endotheliopathy and controls at these time-points in the MD Anderson Cancer Center data, possibly due to analysis and assay differences. Several biomarkers with the highest predictive value at Day 7, including Angiopoietin-1 (Ang-1) and tissue inhibitor of metalloproteinases-1 (TIMP-1) align with the biological pathways highlighted in the St. Jude Children’s Research Center data. The decreased Angiopoietin-1 in endotheliopathy patients highlights an imbalance in the Ang-Tie signaling pathway, resulting in increased vascular permeability and destabilization (21). The Ang-1/Ang-2 ratio has been proposed as a marker of vascular destabilization in post-HSCT patients in prior literature (39) which is consistent with our results. The high predictive value of elevated TIMP-1 further suggests that injury affects both the glycocalyx and the underlying vascular structure, and emphasizes the importance of dysregulated extracellular matrix remodeling in endothelial dysfunction (40). The identification of decreased insulin-like growth factor-binding protein-3 (IGFBP-3) as a predictor of endotheliopathy in the MD Anderson Cancer Center data introduces new opportunities to investigate the influence of growth factors and metabolic signaling (41) on endothelial recovery or failure. Interestingly, both TIMP-1 and IGFBP-3 demonstrated very strong discriminatory ability in this pilot dataset (exploratory AUC of 1) for endotheliopathy not only on Day 7 but also on Day 0. These findings indicate that, even at a very early time point, a subgroup of patients at increased risk of endothelial injury could be identified.

### 4.1 Limitations and methodological notes

The interpretation of our findings must consider certain limitations. The highly complex, dynamic nature of the glycocalyx means that individual patient factors may influence biomarker levels. A key challenge in the St. Jude Children’s Research Hospital Study was matching control biomarker levels to the variable timing of diagnosis in cases. We chose Day 14 in controls as a practical, physiologically relevant comparison, representing a stable post-engraftment period with fewer early inflammatory confounders. Reassuringly, sensitivity testing using Day 7 controls yielded nearly identical results, reinforcing the strength of our main findings and suggesting that earlier measurements are also helpful for risk assessment. It should be noted that Ang-2 was not found to be significantly different between cases of endotheliopathy and controls at Days 0 and 7 in the MD Anderson data. Generalizability of our findings must be confirmed with larger studies. For example, St. Jude Children’s Research Study cohort contained a relatively large proportion of patients who received transplants from haploidentical donors. The MD Anderson Cancer Center cohort contained a relatively large proportion of patients who received cord blood grafts and a majority received myeloablative conditioning. There are additional factors not controlled for such as different specific conditioning regimen and GVHD prophylaxis strategies in individual patients that may affect results. As both studies represent pilot studies with small sample sizes, results need validation in larger, multicenter studies to confirm findings and generalizability of results, and to refine cutoff values and predictive models.

### 4.2 Conclusions and future perspectives

The convergence of these two prospective pilot studies, one targeted and one unbiased, affirms the central role of endothelial injury in post-HSCT complications and provides a list of potential candidate predictors related to endothelial glycocalyx structure and function. Future research should determine whether a single biomarker (Syndecan-1, Ang-2), a combination of markers, or a broader panel (including Ang-1, TIMP-1 and IGFBP-3) best balances predictive accuracy, cost, and feasibility in clinical settings. The strong and consistent predictive power of Syndecan-1 and Ang-2 for detecting endotheliopathy is very promising. Early identification of patients at high risk for endotheliopathy could transform post-HSCT care. We propose that serial early measurements may enable proactive risk management, for example earlier modification of immune suppression (e.g. calcineurin inhibitors). This could allow closer monitoring, earlier diagnosis, and timely treatment with agents such as defibrotide for SOS/VOD or eculizumab for TA-TMA. Given small sample size and generalizability concerns with single center cohorts, future multi-center prospective trials are necessary to validate these findings, optimize measurement timing and cut-off points, and confirm the prognostic role of biomarkers of glycocalyx damage and dysregulation for endotheliopathy complications in children and young adults undergoing HSCT.

## Data Availability

The raw data supporting the conclusions of this article will be made available by the authors, without undue reservation.

## Supplementary material

Supplementary Figure 1 Sensitivity analysis showing biomarker levels immediately preceding endotheliopathy diagnosis **(A)** or SOS/VOD or TA-TMA diagnosis **(B)** compared to Days 7 and 21 in controls. The center line represents the median and the box represents the interquartile range. Whiskers show biomarker levels within 1.5 times the interquartile range and individual points are outliers. For cases, the time-point immediately preceding the diagnosis of either SOS/VOD or TA-TMA was used. For controls, the standardized time-point of Day 14 was used. The figure shows log10 transformation of biomarker levels in pg/mL.

Supplementary Table 1 Individual patient characteristics for St. Jude Children’s Research Hospital study. Abbreviations: anti-thymocyte globulin (ATG), autologous (auto), aplastic anemia (AA), acute lymphoblastic leukemia (ALL), acute myeloid leukemia (AML), bone marrow (BM), capillary leak syndrome (CLS), chronic myeloid leukemia (CML), Diamond-Blackfan anemia (DBA), diffuse alveolar hemorrhage (DAH), engraftment syndrome (ES), gastrointestinal graft versus host disease (GI GVHD), matched sibling donor (MSD), matched unrelated donor (MUD), mismatched related donor (MMRD), mycophenalate mofetil (MMF), myeloablative conditioning (MAC), neuroblastoma (NB), non-myeloablative conditioning (NMA), peripheral blood (PB), reduced intensity conditioning (RIC), sickle cell disease (SCD), sinusoidal obstruction syndrome/veno-occlusive disease (SOS/VOD), total body irradiation (TBI), transplant-associated thrombotic microangiopathy (TA-TMA).

Supplementary Table 2 Individual patient characteristics for MD Anderson Cancer Center study. Abbreviations: anti-thymocyte globulin (ATG), autologous (auto), acute lymphoblastic leukemia (ALL), acute myeloid leukemia (AML), bone marrow (BM), matched sibling donor (MSD), matched unrelated donor (MUD), mismatched related donor (MMRD), mycophenalate mofetil (MMF), myeloablative conditioning (MAC), peripheral blood (PB), post-transplant cyclophosphamide (PTCY), reduced intensity conditioning (RIC), sinusoidal obstruction syndrome/veno-occlusive disease (SOS/VOD), transplant-associated thrombotic microangiopathy (TA-TMA), total body irradiation (TBI), umbilical cord blood (UCB).

Supplementary Table 3 MD Anderson Cancer Center study exploratory AUC, threshold, sensitivity, and specificity at Day 0 for later development of endotheliopathy.

Supplementary Table 4 MD Anderson Cancer Center study exploratory AUC, threshold, sensitivity, and specificity at Day 0 for later development of endotheliopathy.
