# Supplementary figures and images for: Glycocalyx biomarkers as early predictors of endotheliopathy in pediatric and young adult hematopoietic stem cell transplantation patients
Source: Front Oncol. 2026 May 8;16:1789000. doi: 10.3389/fonc.2026.1789000 (PMC13193815; doi:10.3389/fonc.2026.1789000)

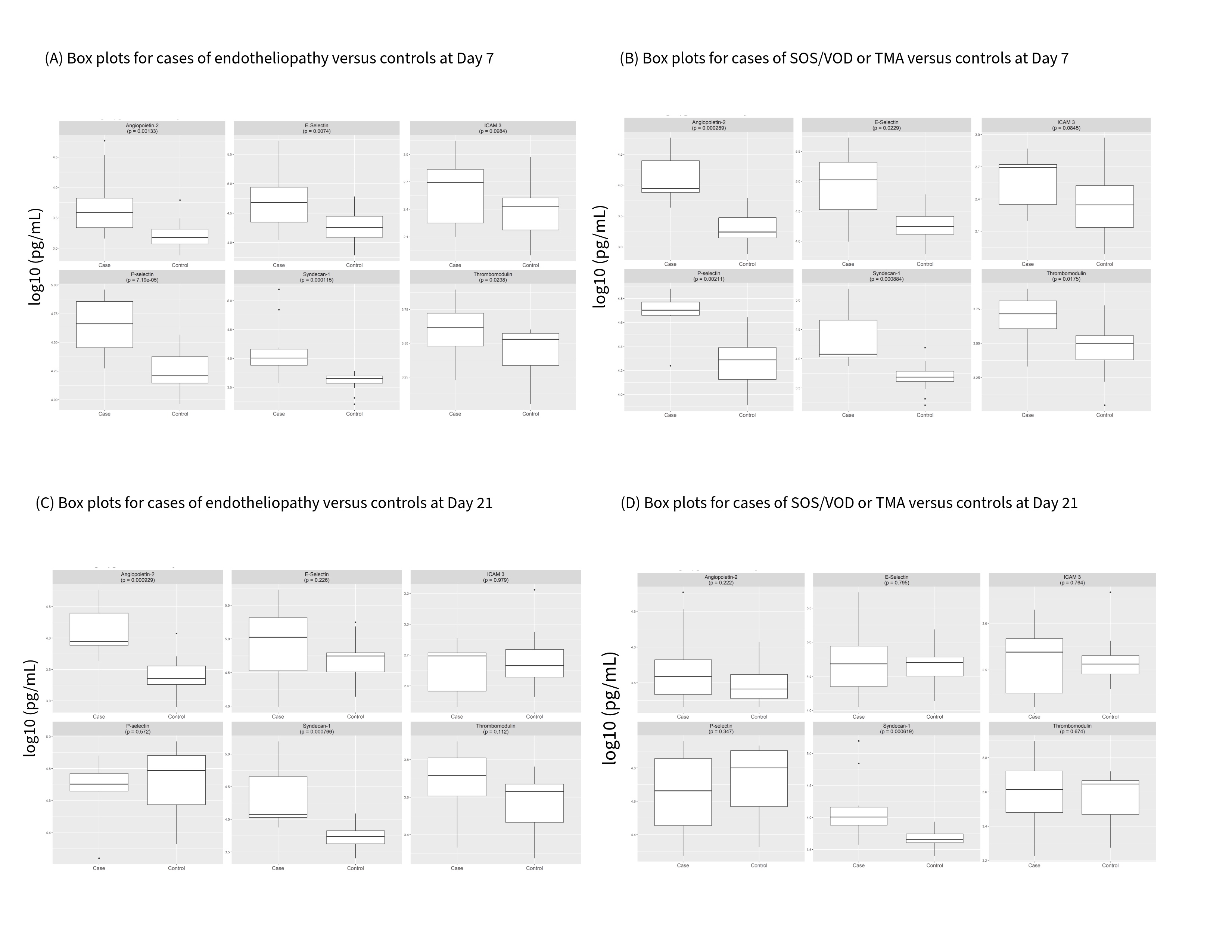

Supplement: Supplementary Figure 1 — Sensitivity analysis showing biomarker levels immediately preceding endotheliopathy diagnosis (A) or SOS/VOD or TA-TMA diagnosis (B) compared to Days 7 and 21 in controls. The center line represents the median and the box represents the interquartile range. Whiskers show biomarker levels within 1.5 times the interquartile range and individual points are outliers. For cases, the time-point immediately preceding the diagnosis of either SOS/VOD or TA-TMA was used. For controls, the standardized time-point of Day 14 was used. The figure shows log10 transformation of biomarker levels in pg/mL. [file Image1.jpeg]
