# Supplementary material for: Glycocalyx biomarkers as early predictors of endotheliopathy in pediatric and young adult hematopoietic stem cell transplantation patients
Source: Front Oncol. 2026 May 8;16:1789000. doi: 10.3389/fonc.2026.1789000 (PMC13193815; doi:10.3389/fonc.2026.1789000)
Supplement: Supplementary Table 1 — Individual patient characteristics for St. Jude Children’s Research Hospital study. Abbreviations: anti-thymocyte globulin (ATG), autologous (auto), aplastic anemia (AA), acute lymphoblastic leukemia (ALL), acute myeloid leukemia (AML), bone marrow (BM), capillary leak syndrome (CLS), chronic myeloid leukemia (CML), Diamond-Blackfan anemia (DBA), diffuse alveolar hemorrhage (DAH), engraftment syndrome (ES), gastrointestinal graft versus host disease (GI GVHD), matched sibling donor (MSD), matched unrelated donor (MUD), mismatched related donor (MMRD), mycophenalate mofetil (MMF), myeloablative conditioning (MAC), neuroblastoma (NB), non-myeloablative conditioning (NMA), peripheral blood (PB), reduced intensity conditioning (RIC), sickle cell disease (SCD), sinusoidal obstruction syndrome/veno-occlusive disease (SOS/VOD), total body irradiation (TBI), transplant-associated thrombotic microangiopathy (TA-TMA). [file Table1.docx]

Supplemental Table 1. Individual patient characteristics for St. Jude Children’s Research Hospital study. Abbreviations: anti-thymocyte globulin (ATG), autologous (auto), aplastic anemia (AA), acute lymphoblastic leukemia (ALL), acute myeloid leukemia (AML), bone marrow (BM), capillary leak syndrome (CLS), chronic myeloid leukemia (CML), Diamond-Blackfan anemia (DBA), diffuse alveolar hemorrhage (DAH), engraftment syndrome (ES), gastrointestinal graft versus host disease (GI GVHD), matched sibling donor (MSD), matched unrelated donor (MUD), mismatched related donor (MMRD), mycophenalate mofetil (MMF), myeloablative conditioning (MAC), neuroblastoma (NB), non-myeloablative conditioning (NMA), peripheral blood (PB), reduced intensity conditioning (RIC), sickle cell disease (SCD), sinusoidal obstruction syndrome / veno-occlusive disease (SOS/VOD), total body irradiation (TBI), transplant-associated thrombotic microangiopathy (TA-TMA).

| Age at transplant (years) | Sex | History of prior transplant | Transplant indication | Donor type | Graft type | Conditioning regimen | Conditioning Agents | T cell depletion, if applicable | GVHD Prophylaxis Agents | Post-HSCT Endotheliopathy type(s) developed, if applicable | Day of post-HSCT endotheliopathy diagnosis, if applicable | Reason(s) for ICU admission, if applicable | Day of ICU admission, if applicable |
| --- | --- | --- | --- | --- | --- | --- | --- | --- | --- | --- | --- | --- | --- |
| 13 | M | Yes | AML | MUD | PB | NMA | N/A | ATG | Cyclosporine, Methotrexate | N/A | N/A | NA | N/A |
| 8 | F | No | NB | Auto | PB | RIC | Busulfan, Melphalan | N/A | None | N/A | N/A | Septic shock | Day 79 |
| 19 | M | No | ALL | MMRD | PB | RIC | Cyclophosphamide, Melphalan, Fludarabine, Thiotepa | ATG | None | N/A | N/A | N/A | N/A |
| 16 | M | No | AA | MMRD | BM | RIC | Melphalan, Fludarabine, Thiotepa | ATG | Cyclosporine, Methotrexate, Tacrolimus | N/A | N/A | N/A | N/A |
| 9 | M | No | SCD | MSD | PB | RIC | Thiotepa | Alemtuzumab | Sirolimus | N/A | N/A | N/A | N/A |
| 22 | F | No | AML | MMRD | BM | MAC | Busulfan, Fludarabine, Thiotepa | N/A | Cyclophosphamide, MMF, Tacrolimus | N/A | N/A | N/A | N/A |
| 7 | F | No | NB | Auto | PB | MAC | Cyclophosphamide, Thiotepa | N/A | None | CLS | Day 7 | N/A | N/A |
| 18 | F | No | SCD | MMRD | PB | RIC | TBI, Thiotepa | N/A | Sirolimus, Cyclophosphamide | TA-TMA | Day 94 | N/A | N/A |
| 17 | M | No | AA | MUD | PB | RIC | Busulfan | ATG | Ruxolitinib, Sirolimus, Cyclophosphamide, Tacrolimus, MMF, Steroids | N/A | N/A | Shock | Day 80 |
| 8 | F | No | DBA | MUD | BM | RIC | Melphalan, Fludarabine, Rituximab Thiotepa | ATG | Cyclosporine, Methotrexate, Tacrolimus | N/A | N/A | N/A | N/A |
| 16 | F | No | Germ cell tumor | Auto | PB | MAC | Carboplatin, Etoposide | N/A | None | N/A | N/A | N/A | N/A |
| 3 | F | No | AML | MMRD | PB | RIC | Cyclophosphamide, Melphalan, Fludarabine, Thiotepa | ATG | None | ES | Day 11 | N/A | N/A |
| 9 | M | Yes | ALL | MMRD | PB | RIC | Cyclophosphamide, Melphalan, Fludarabine, Rituximab, Thiotepa | ATG | Sirolimus | ES | Day 12 | Pericardial effusion | Day 69 |
| 14 | F | Yes | AML | MMRD | PB | RIC | Cyclophosphamide, Melphalan, Fludarabine, Rituximab, Thiotepa | ATG | Sirolimus | ES | Day 14 | N/A | N/A |
| 6 | M | No | ALL | MMRD | PB | RIC | Cyclophosphamide, Melphalan, Fludarabine, Thiotepa | ATG | None | Gastrointestinal GVHD | Day 72 | N/A | N/A |
| 9 | M | Yes | AML | MUD | PB | MAC | Cyclophosphamide, TBI | ATG | Cyclosporine, Methotrexate, Sirolimus, Steroids | CLS; TA-TMA; SOS/VOD | Day 9 (CLS, SOS/VOD)  Day 21 (TA-TMA) | Respiratory failure | Day 9 |
| 3 | M | No | ALL | MSD | BM | MAC | Cyclophosphamide | N/A | Tacrolimus, Cyclosporine, MMF | CLS | Day 10 | Septic shock | Day 54 |
| 12 | F | No | AML | MMRD | PB | RIC | Cyclophosphamide, Melphalan, Fludarabine, Thiotepa | ATG | None | N/A | N/A | N/A | N/A |
| 2 | M | No | AML | MMRD | PB | RIC | Cyclophosphamide, Melphalan, Fludarabine, Thiotepa | ATG | MMF | ES, SOS/VOD | Day 21 (ES)  Day 63 (SOS/VOD) | N/A | N/A |
| 6 | M | No | CML | MSD | BM | MAC | Busulfan, Cyclophosphamide | N/A | Cyclosporine, MMF | TA-TMA, SOS/VOD | Day 21  (SOS/VOD)  Day 21 (TA-TMA) | N/A | N/A |
| 16 | M | No | AML | MMRD | PB | RIC | Melphalan, Fludarabine, Thiotepa | N/A | Cyclophosphamide, Tacrolimus, MMF | CLS | Day 7 | N/A | N/A |
| 4 | M | Yes | AML | MMRD | PB | RIC | Cyclophosphamide, Melphalan, Fludarabine, Rituximab, Thiotepa | ATG | Sirolimus | N/A | N/A | N/A | N/A |
| 11 | F | No | ALL | MMRD | PB | RIC | Cyclophosphamide, Melphalan, Fludarabine, Rituximab, Thiotepa | ATG | Sirolimus, Ruxolitinib | TA-TMA; GI GVHD | Day 11 (TA-TMA)  Day 88 (GI GVHD) | Respiratory failure, renal failure | Day 11 |
| 14 | M | No | AA | MMRD | PB | RIC | Cyclophosphamide, Fludarabine | ATG | Cyclophosphamide, Tacrolimus, MMF | CLS | Day 1 | N/A | N/A |
| 21 | F | No | AA | MUD | BM | MAC | Busulfan, Cyclophosphamide | ATG | Methotrexate | N/A | N/A | N/A | N/A |
| 15 | F | No | ALL | MUD | BM | MAC | Cyclophosphamide | ATG | Cyclosporine, Methotrexate, Steroids | TA-TMA; SOS/VOD; DAH | Day 10 (SOS/VOD)  Day 10 (TA-TMA)  Day 36 (DAH) | Respiratory failure | Day 34 |
| 14 | M | No | AML | MMRD | PB | RIC | Cyclophosphamide, Melphalan, Fludarabine, Rituximab, Thiotepa | ATG | Sirolimus | N/A | N/A | N/A | N/A |
| 4 | M | No | AML | MMRD | PB | RIC | Cyclophosphamide, Melphalan, Fludarabine, Thiotepa | N/A | Tacrolimus, MMF | N/A | N/A | N/A | N/A |
| 2 | M | No | AML | MMRD | PB | RIC | Melphalan, Fludarabine, Thiotepa | N/A | Cyclophosphamide, Tacrolimus, MMF | ES | Day 4 | N/A | N/A |
| 13 | M | No | CML | MUD | BM | MAC | Busulfan, Cyclophosphamide | ATG | Methotrexate, Cyclosporine | N/A | N/A | N/A | N/A |
| 5 | F | No | T-cell lymphoma | MMRD | PB | NMA | Fludarabine | N/A | Cyclophosphamide, MMF, Tacrolimus | N/A | N/A | N/A | N/A |
