# Supplementary material for: Glycocalyx biomarkers as early predictors of endotheliopathy in pediatric and young adult hematopoietic stem cell transplantation patients
Source: Front Oncol. 2026 May 8;16:1789000. doi: 10.3389/fonc.2026.1789000 (PMC13193815; doi:10.3389/fonc.2026.1789000)
Supplement: Supplementary Table 2 — Individual patient characteristics for MD Anderson Cancer Center study. Abbreviations: anti-thymocyte globulin (ATG), autologous (auto), acute lymphoblastic leukemia (ALL), acute myeloid leukemia (AML), bone marrow (BM), matched sibling donor (MSD), matched unrelated donor (MUD), mismatched related donor (MMRD), mycophenalate mofetil (MMF), myeloablative conditioning (MAC), peripheral blood (PB), post-transplant cyclophosphamide (PTCY), reduced intensity conditioning (RIC), sinusoidal obstruction syndrome/veno-occlusive disease (SOS/VOD), transplant-associated thrombotic microangiopathy (TA-TMA), total body irradiation (TBI), umbilical cord blood (UCB). [file Table2.docx]

Supplemental Table 2. Individual patient characteristics for MD Anderson Cancer Center study. Abbreviations: anti-thymocyte globulin (ATG), autologous (auto), acute lymphoblastic leukemia (ALL), acute myeloid leukemia (AML), bone marrow (BM), matched sibling donor (MSD), matched unrelated donor (MUD), mismatched related donor (MMRD), mycophenalate mofetil (MMF), myeloablative conditioning (MAC), peripheral blood (PB), post-transplant cyclophosphamide (PTCY), reduced intensity conditioning (RIC), sinusoidal obstruction syndrome / veno-occlusive disease (SOS/VOD), transplant-associated thrombotic microangiopathy (TA-TMA), total body irradiation (TBI), umbilical cord blood (UCB).

| Age at transplant (years) | Sex | History of prior transplant | Transplant indication | Donor type | Graft type | Conditioning regimen | Conditioning Agents | T cell depletion, if applicable | GVHD Prophylaxis Agents | Post-HSCT Endotheliopathy type(s) developed, if applicable | Day of post-HSCT endotheliopathy diagnosis, if applicable | Reason(s) for ICU admission, if applicable | Day of ICU admission, if applicable |
| --- | --- | --- | --- | --- | --- | --- | --- | --- | --- | --- | --- | --- | --- |
| 8 | F | No | Mucopolysaccharidosis | Unrelated UCB | Single cord blood unit | MAC | Busulfan, Cyclophosphamide | ATG | MMF,  Tacrolimus | SOS/VOD, DAH, GI GVHD,TMA | Day 13 (SOS/VOD)  37 (TMA)  Day 62 (GI GVHD)  D 76 (DAH) | Respiratory failure, shock | Day 6 |
| 2 | M | No | AML | Unrelated UCB | Single cord blood unit | MAC | Busulfan, Cyclophosphamide | ATG | MMF,  Tacrolimus | SOSVOD | Day 9 (SOS/VOD) | N/A | N/A |
| 20 | M | No | ALL | Unrelated Double UCB | Double cord blood unit | MAC | Busulfan, Clofarabine, Fludarabine, TBI | ATG | MMF,  Tacrolimus | SOS/VOD, GI GVHD | Day 7 (SOS/VOD)  D 121 (GI GVHD) | Respiratory failure, shock | Day 2 |
| 5 | M | No | ALL | Unrelated UCB | Single cord blood unit | MAC | Cyclophosphamide, Fludarabine, TBI | N/A | MMF,  Tacrolimus | SOS/VOD, GI GVHD | Day 13 (SOS/VOD)  D 27 (GI GVHD) | Renal failure | Day 15 |
| 6 | F | No | AML | MUD | PB | MAC | Busulfan, Fludarabine | PTCY | MMF,  Tacrolimus | N/A | N/A | N/A | N/A |
| 23 | F | No | Hodgkin Lymphoma | Autologous | PB | MAC | Busulfan, Gemcitabine, Melphalan, SAHA | N/A | N/A | N/A | N/A | N/A | N/A |
| 11 | M | No | AML | MSD | BM | MAC | Busulfan, Fludarabine | PTCY | MMF,  Tacrolimus | N/A | N/A | N/A | N/A |
| 18 | M | No | AML | MUD | PB | MAC | Busulfan, Fludarabine | PTCY | MMF,  Tacrolimus | N/A | N/A | N/A | N/A |
| 24 | F | No | AML | MSD | PB | MAC | Busulfan, Fludarabine, Sorafenib | PTCY | MMF,  Tacrolimus | N/A | N/A | N/A | N/A |
| 21 | M | Yes | Blastic plasmacytoid dendritic cell neoplasm | MMRD | BM | RIC | Fludarabine, Melphalan, TBI | PTCY | MMF,  Tacrolimus | N/A | N/A | N/A | N/A |
| 23 | M | No | Ewing Sarcoma | Autologous | PB | MAC | Busulfan, Melphalan, Topotecan | N/A | N/A | N/A | N/A | N/A | N/A |
| 20 | F | No | Hodgkin Lymphoma | Autologous | PB | MAC | Busulfan, Gemcitabine, Melphalan, SAHA | N/A | N/A | N/A | N/A | N/A | N/A |
| 25 | F | No | Hodgkin Lymphoma | Autologous | PB | MAC | Busulfan, Gemcitabine, Melphalan, SAHA | N/A | N/A | N/A | N/A | N/A | N/A |
