# Supplementary material for: Glycocalyx biomarkers as early predictors of endotheliopathy in pediatric and young adult hematopoietic stem cell transplantation patients
Source: Front Oncol. 2026 May 8;16:1789000. doi: 10.3389/fonc.2026.1789000 (PMC13193815; doi:10.3389/fonc.2026.1789000)
Supplement: Supplementary Table 4 — MD Anderson Cancer Center study exploratory AUC, threshold, sensitivity, and specificity at Day 0 for later development of endotheliopathy. [file Table4.docx]

Supplemental Table 4. MD Anderson Cancer Center study exploratory AUC, threshold, sensitivity, and specificity at Day 0 for later development of endotheliopathy.

| Biomarker | AUC | Youden Index | | | |
| --- | --- | --- | --- | --- | --- |
|  |  |  |  |  |  |
|  |  | Threshold | Sensitivity | Specificity |  |
| Activin A | 0.861 | 0.39 | 0.67 | 1 |  |
| ADAMTS-1 | 0.833 | 0.39 | 0.67 | 1 |  |
| Amphiregulin | 0.889 | 0.37 | 0.67 | 1 |  |
| Angiogenin | 0.778 | 0.96 | 0.56 | 1 |  |
| Angiopoietin-1 | 0.972 | 0.34 | 0.89 | 1 |  |
| Angiopoietin-2 | 0.722 | 0.55 | 0.56 | 1 |  |
| Angiostatin/Plasminogen | 0.861 | 0.38 | 0.67 | 1 |  |
| Artemin | 0.861 | 0.37 | 0.67 | 1 |  |
| Coagulation Factor III | 0.944 | 0.35 | 0.78 | 1 |  |
| CXCL16 | 0.75 | 0.63 | 0.56 | 1 |  |
| DPPIV | 0.611 | 0.87 | 1 | 0.5 |  |
| EGF | 0.833 | 0.51 | 0.56 | 1 |  |
| EG-VEGF | 0.944 | 0.35 | 0.78 | 1 |  |
| Endoglin | 0.75 | 0.54 | 0.67 | 1 |  |
| Endostatin/Collagen XVIII | 0.639 | 0.65 | 0.56 | 1 |  |
| Endothelin-1 | 0.75 | 0.51 | 0.56 | 1 |  |
| FGF acidic | 0.806 | 0.5 | 0.56 | 1 |  |
| FGF basic | 0.889 | 0.35 | 0.78 | 1 |  |
| FGF-4 | 0.944 | 0.35 | 0.78 | 1 |  |
| FGF-7 | 0.75 | 0.51 | 0.56 | 1 |  |
| GDNF | 0.889 | 0.37 | 0.67 | 1 |  |
| GM-CSF | 0.944 | 0.35 | 0.78 | 1 |  |
| HB-EGF | 0.806 | 0.53 | 0.56 | 1 |  |
| HGF | 0.806 | 0.39 | 0.67 | 1 |  |
| IGFBP-1 | 0.806 | 0.79 | 0.89 | 0.75 |  |
| IGFBP-2 | 0.833 | 0.92 | 1 | 0.75 |  |
| IGFBP-3 | 1 | 0.65 | 1 | 1 |  |
| IL-1beta | 0.778 | 0.51 | 0.56 | 1 |  |
| IL-8 | 0.806 | 0.51 | 0.56 | 1 |  |
| LAP (TGF-beta1) | 0.861 | 0.38 | 0.67 | 1 |  |
| Leptin | 0.833 | 0.52 | 0.67 | 1 |  |
| MCP-1 | 0.889 | 0.51 | 0.67 | 1 |  |
| MIP-1a | 0.833 | 0.39 | 0.67 | 1 |  |
| MMP-8 | 0.833 | 0.41 | 0.67 | 1 |  |
| MMP-9 | 0.833 | 0.66 | 0.56 | 1 |  |
| NC | 0.694 | 0.54 | 0.56 | 1 |  |
| NRG1-beta1 | 0.861 | 0.4 | 0.67 | 1 |  |
| PD-ECGF | 0.944 | 0.39 | 0.78 | 1 |  |
| PDGF-AA | 0.833 | 0.39 | 0.67 | 1 |  |
| PDGF-AB/PDGF-BB | 0.917 | 0.35 | 0.78 | 1 |  |
| Pentraxin 3 (PTX3) | 0.778 | 0.8 | 0.89 | 0.75 |  |
| Persephin | 0.75 | 0.51 | 0.56 | 1 |  |
| Platelet Factor 4 (PF4) | 0.472 | 0.76 | 0.33 | 1 |  |
| PlGF | 0.861 | 0.38 | 0.67 | 1 |  |
| Prolactin | 0.806 | 0.48 | 0.67 | 1 |  |
| Serpin B5 | 0.833 | 0.39 | 0.67 | 1 |  |
| Serpin E1 | 0.611 | 0.72 | 0.56 | 1 |  |
| Serpin F1 | 0.667 | 0.59 | 0.56 | 1 |  |
| Thrombospondin-1 | 0.778 | 0.93 | 1 | 0.75 |  |
| Thrombospondin-2 | 0.833 | 0.39 | 0.67 | 1 |  |
| TIMP-1 | 1 | 0.98 | 1 | 1 |  |
| TIMP-4 | 0.722 | 0.61 | 0.56 | 1 |  |
| uPA | 0.889 | 0.37 | 0.67 | 1 |  |
| Vasohibin | 0.889 | 0.37 | 0.67 | 1 |  |
| VEGF | 0.833 | 0.4 | 0.67 | 1 |  |
| VEGF-C | 0.833 | 0.38 | 0.67 | 1 |  |
